# Benefits of chronic total coronary occlusion percutaneous intervention in patients with heart failure and reduced ejection fraction: insights from a cardiovascular magnetic resonance study

**DOI:** 10.1186/s12968-016-0287-5

**Published:** 2016-11-05

**Authors:** Montserrat Cardona, Victoria Martín, Susanna Prat-Gonzalez, José Tomás Ortiz, Rosario Jesús Perea, Teresa Maria de Caralt, Mónica Masotti, Félix Pérez-Villa, Manel Sabaté

**Affiliations:** 1 Department of Cardiology, Cardiovascular Institute, Hospital Clínic de Barcelona, Institute of Biomedical Research August Pi i Sunyer (IDIBAPS), Villarroel 170, 08036 Barcelona, Spain; 2 Department of Radiodiagnosis, Centro de Diagnóstico por la Imagen, Hospital Clínic de Barcelona, Institute of Biomedical Research August Pi i Sunyer (IDIBAPS), Barcelona, Spain

**Keywords:** Angioplasty, Cardiovascular magnetic resonance, Chronic total coronary occlusion, Heart failure with reduced ejection fraction, Myocardial viability

## Abstract

**Background:**

Chronic total occlusion percutaneous coronary intervention (CTO-PCI) can improve angina and left ventricular ejection fraction (LVEF). These benefits were not assessed in populations with heart failure with reduced ejection fraction (HFrEF). We studied the effect of CTO-PCI on left ventricular function and clinical parameters in patients with HFrEF.

**Methods:**

Using cardiovascular magnetic resonance (CMR), we studied 29 patients with HFrEF and evidence of viability and/or ischemia in the territory supplied by a CTO who were successfully treated with CTO-PCI. In patients with multi-vessel disease, non-CTO PCI was also performed. Imaging parameters, clinical status, and brain natriuretic peptide (BNP) levels were evaluated before and 6 months after CTO-PCI.

**Results:**

A decrease in left ventricular end-systolic volume (160 ± 54 ml vs. 143 ± 58 ml; *p* = 0.029) and an increase in LVEF (31.3 ± 7.4 % vs. 37.7 ± 8 %; *p* < 0.001) were observed. There were no differences in LVEF improvement between patients who underwent non-CTO PCI (*n* = 11) and those without this intervention (*n* = 18); (*p* = 0.73). The number of segments showing perfusion defects was significantly reduced (0.5 ± 1 vs. 0.2 ± 0.5; *p* = 0.043). Angina (*p* = 0.002) and NYHA functional class (*p* = 0.004) improved, and BNP levels decreased (*p* = 0.004) after CTO-PCI.

**Conclusions:**

In this group of patients with HFrEF showing CMR evidence of viability and/or ischemia within the territory supplied by the CTO, an improvement in ejection fraction, left ventricular end-systolic volume and ischemia burden was observed after CTO-PCI. Clinical and laboratory parameters also improved.

**Trial registration:**

ClinicalTrials.gov NCT02570087. Registered 6 October 2015.

## Background

A chronic total occlusion (CTO) is defined as a coronary obstruction with thrombolysis in myocardial infarction (TIMI) grade 0 flow that persists for at least 3 months [1]. This type of lesions can be found in up to 50 % of patients with significant coronary disease on angiography [2]. CTO percutaneous coronary intervention (PCI) is performed infrequently likely due to technical complexity, the potential for major periprocedural complications, the relatively low procedural success rates and controversial data regarding the clinical benefit [3, 4]. According to consensus documents CTO-PCI should be considered in the presence of symptoms (or objective evidence of a large region of ischemia/viability) when the myocardium supplied by the CTO is viable, the likelihood of success is >60 % and the anticipated major complication rate is low [5, 6]. Potential CTO-PCI benefits are ischemia reduction, angina relief, and improved left ventricular ejection function (LVEF) and long-term survival [7–18]. However, these data come from studies enrolling patients with preserved LVEF, where clinical benefits of myocardial revascularization are potentially lower. Thus, the present study aimed to assess whether CTO-PCI in patients with chronic heart failure and reduced ejection fraction (HFrEF) is associated with an improvement in LVEF, angina status, New York Heart Association (NYHA) functional class for dyspnea (NYHA I-IV) and brain natriuretic peptide (BNP) levels.

## Methods

### Study population

**Fig. 1:**
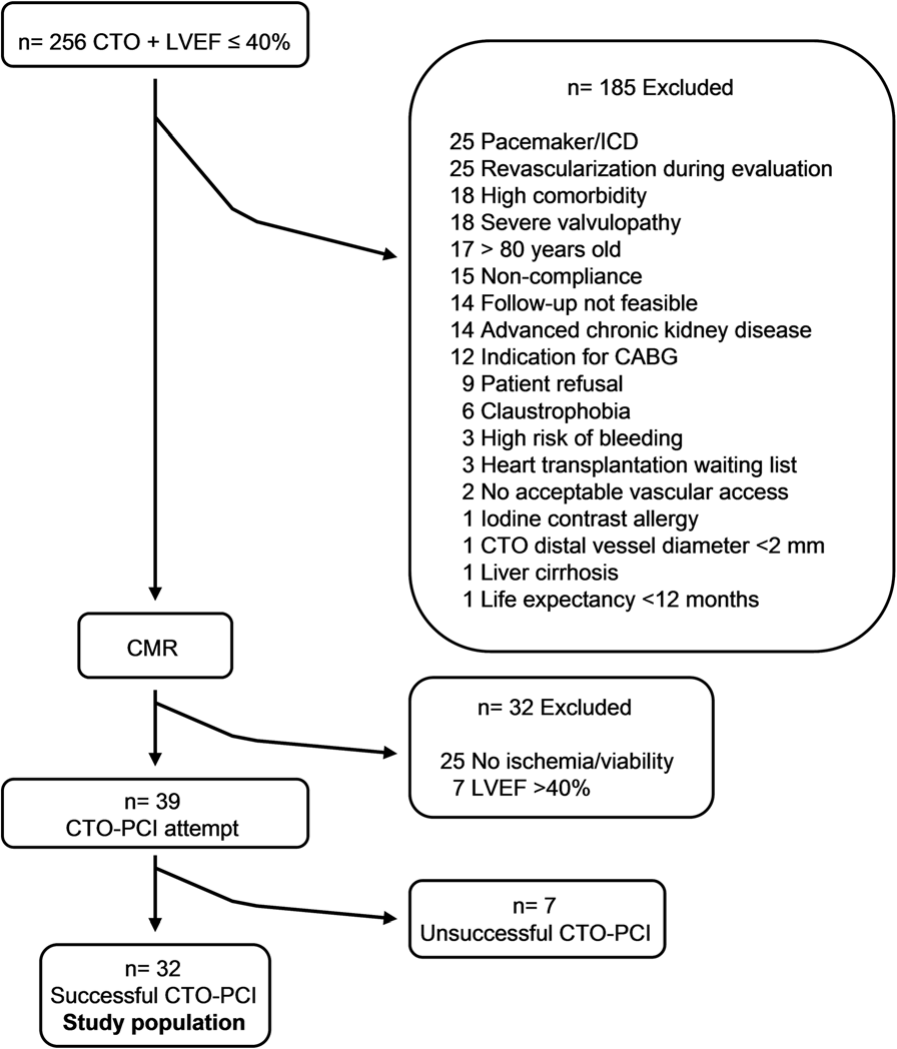
Flow chart of patient inclusion. Abbreviations: CABG, coronary artery bypass graft; CMR, cardiovascular magnetic resonance; CTO, chronic total occlusion; ICD, implantable cardioverter defibrillator; LVEF, left ventricular ejection fraction; PCI, percutaneous coronary intervention

We prospectively collected data from consecutive patients referred to our institution for invasive coronary angiography from January 2011 to June 2013, selecting those with at least one CTO and LVEF ≤40 % measured by echocardiography, gated-SPECT or contrast ventriculography (*n* = 256). A CTO was defined as an occlusion of at least 3 months of duration based on a previous angiogram showing the occluded vessel or clinical data on previous coronary ischemic events potentially related to a coronary occlusion. Patients who did not meet any of the exclusion criteria, detailed below, underwent cardiovascular magnetic resonance (CMR) study (Fig. 1). CTO recanalization was attempted in those with LVEF ≤40 % confirmed by CMR and with evidence of myocardial viability and/or ischemia in at least two contiguous segments subtended by the CTO (*n* = 39). The main reasons for exclusion were a lack of ischemia and myocardial viability in at least two contiguous CTO dependent myocardial segments by CMR (*n* = 25; 12 %), the presence of a pacemaker or an implantable cardioverter defibrillator (*n* = 25; 12 %) and surgical or percutaneous revascularization during the candidacy evaluation with no chance to perform basal assessments (*n* = 25; 12 %). The final study population included the 32 patients with successful CTO-PCI (82 % success rate).

### Exclusion criteria

Clinical criteria for exclusion were women of childbearing age or pregnant, patients younger than 18 or older than 80 years, contraindications for CMR (pacemakers, implantable cardioverter defibrillators, claustrophobia and cochlea implants), or hospital admission within the previous 90 days due to decompensated heart failure, myocardial infarction or unstable angina. Additional criteria were iodine contrast or gadolinium allergy, aspirin or clopidogrel allergy, asthma, NYHA IV, chronic kidney disease with serum creatinine levels ≥2.5 mg/dl or glomerular filtration rate ≤30 ml/min/1.73 m^2^, severe valvulopathy, indication for coronary artery bypass surgery, heart transplantation waiting list, high comorbidity, liver cirrhosis, evidence of active bleeding or high risk of bleeding, follow-up not feasible, noncompliance with medical treatment or life expectancy shorter than 12 months.

Technical criteria for exclusion were CTO distal vessel not visible through collateral circulation, CTO distal vessel diameter <2 mm or absence of acceptable vascular access.

### CMR studies

All CMR studies (baseline and 6-month follow-up, mean duration of 45 min) were performed using a General Electric Signa HDxt 1.5-T scanner equipped with an 8-channel coil and cardiac-dedicated software. Perfusion studies were conducted using a gradient-echo turbo-field sequence prescribed in the left ventricular short-axis orientation, at the basal, mid-ventricular and apical levels after 4 min of intravenous administration of adenosine (Atepodin®) at a dose of 140 mcg/kg/min and simultaneous administration of 0.1 mmol/kg of gadobutrol (Gadovist®, Bayer Hispania) at 5 ml/s rate. Functional and volumetric assessment of the left ventricle (LV) was conducted by conventional Steady State Free Precession (SSFP) cine sequence, prescribed in sequential short-axis slices, and encompassing the entire LV as well as the 2-, 3-, and 4-chamber views. Typical temporal and in-plane spatial resolution of these images was 40 ms and 1.4 × 1.4 mm, respectively. Rest perfusion images were obtained at least 10 min after the stress perfusion study using the same sequence, location, and contrast injection protocol. At 10 min post-administration of the gadolinium dose for the rest perfusion study, late gadolinium-enhancement (LGE) images were obtained using a segmented inversion-recovery spoiled gradient echo sequence in the same location and identical spatial resolution as the cine images.

### Image analysis

**Fig. 2:**
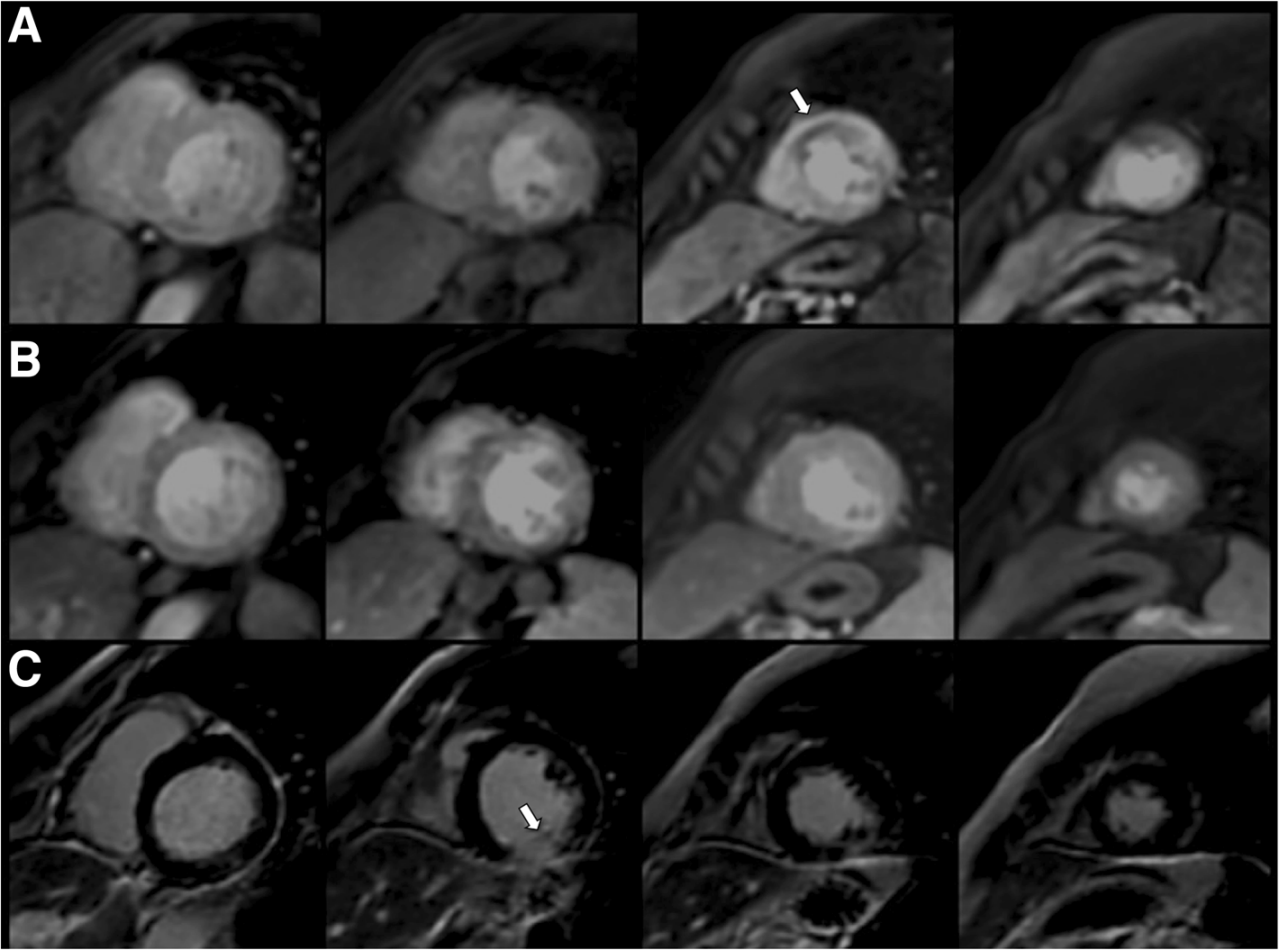
CMR study before PCI in a patient with a CTO in the mid-LAD coronary artery and no other significant coronary stenosis, but a history of previous inferior ST-elevation myocardial infarction with mid RCA stent implantation. **a** Stress perfusion images showing an inducible defect in mid-apical anterior and anteroseptal segments (*white arrow*). **b** Resting perfusion images showing no perfusion defects. **c** LGE study showing transmural enhancement in mid-inferior segment (>75 % transmural extension) (*white arrow*). Absence of enhancement in basal, mid, and apical antero-septal segments

In order to calculate LVEF, LV mass and left ventricular end-systolic (LVESV) and end-diastolic (LVEDV) volumes, the endocardial and epicardial borders were manually traced at end-systole and end-diastole in the cine short-axis images using a dedicated software package (ReportCard, GE). In addition, infarct size was quantified by planimetry of enhanced areas on the stack of short-axis images. Regional wall motion analysis was performed by visual grading of the cine images according to the 17-segment model proposed by the American Heart Association [19] as follows: 0 = normal function, 1 = mild or moderate hypokinesia, 2 = severe hypokinesia, 3 akinesia, 4 = dyskinesia. In myocardial perfusion images, the presence and extent, or absence of a perfusion defect, defined as an evident and maintained hypoperfusion in more than 50 % of the segment seen in the stress images but not in the rest images, were evaluated by visual analysis of myocardial contrast uptake according to a 16-segment model (excluding the 17^th^ segment) (Fig. 2). The presence and transmural extent of LGE was graded in every segment using a 5-point scoring system: absence of LGE = 0, <25 % transmural extent = 1, 26 %–50 % = 2, 51 %–75 % = 3 and >75 % = 4.

Any segment graded ≤2 was considered viable [20]. Image analysis pre and post-PCI was performed by two independent experienced operators masked to the patient’s coronary anatomy and to PCI results; disparities in their evaluation were resolved by consensus with a third independent operator. Appropriate allocation between the involved myocardial segments and the correspondent coronary anatomy in each case was evaluated according to previously reported criteria [21].

### Revascularization strategy

Unilateral or bilateral femoral puncture was the vascular access of choice. Antegrade approach was the choice in all cases, with retrograde approach being used in some re-attempts. With the intention being complete anatomical revascularization, non-CTO PCI was performed when myocardial viability was detected. In the presence of multivessel disease and appropriate distal beds for surgical revascularization, CTO-PCI was the first scheduled interventional procedure enabling patient referral to surgical revascularization in case of failure. If the patient was not a candidate for surgical revascularization, any non-occlusive stenosis was treated first, leaving CTO-PCI as the last procedure. The average time between PCI procedures was 4 weeks. Drug-eluting stents were implanted when feasible. Successful CTO-PCI was defined as the restoration of TIMI grade 3 flow with residual stenosis of less than 30 % and no immediate angiographic complications.

### Clinical follow-up and BNP levels

Clinical follow-up visits were scheduled at 30 days, 3 months and 6 months after CTO-PCI. Angina status and NYHA functional class for dyspnea were assessed before and 6 months after successful CTO-PCI. Plasma levels of BNP were also obtained before the procedure and at follow-up using ADVIA Centaur® BNP chemiluminescence immunoassay and autoanalyzer (Siemens Healthcare Diagnostics). All patients included in the study had been on guidelines recommended medication for HFrEF for at least 3 months before CTO-PCI procedure. Furthermore, during the 6-month study period there were no changes in prescribed medical treatment for angina or heart failure according to data collected at each follow-up visit, and no cardiac resynchronization therapy devices were implanted.

### Statistical analysis

In the descriptive analysis, categorical variables are presented as n and percentage. Continuous variables are presented as mean ± SD when normally distributed, and median (interquartile range [IQR]) otherwise. Group differences were evaluated using chi-square or Fisher exact test for categorical variables. Student t test and Mann-Whitney rank sum test were used for continuous variables. Comparisons between pre- and post-PCI clinical parameters, BNP levels, and CMR studies were performed by paired t-tests, Wilcoxon signed rank or McNemar test, as appropriate. CMR regional contractility and perfusion defect analysis were repeated, taking into account only those segments within the myocardium subtended by a CTO considered for PCI. Additionally, the difference in LVEF between baseline and 6-month follow-up CMR studies was analyzed in a mixed-effects multivariate model adjusted for baseline characteristics and non-CTO PCI procedure. A two-tailed *p* < 0.05 was considered significant. Statistical analysis was performed using SPSS version 13.0 (SPSS, Inc.; Chicago, Illinois, USA).

## Results

### Study population

In total, 32 patients with successful CTO-PCI were included in the study. Baseline demographic and clinical characteristics of this population are shown in Table 1.

**Table 1:** Demographic and clinical characteristics of the study group

	Successful CTO-PCI *n* = 32
Age (y)	59 ± 10.2
Female	8 (21 %)
Hypertension	15 (47 %)
Hyperlipidemia	17 (53 %)
Diabetes	10 (31 %)
HbA1C (%)	7 ± 1.7
History of smoking	
No	13 (41 %)
Yes	11 (34 %)
Current smoker	8 (25 %)
Creatinine (mg/dl)	1 ± 0.2
GFR (MDRD-4; ml/min)	58 ± 5.2
Previous stroke	1 (3 %)
Peripheral arterial vasculopathy	8 (25 %)
LVEF (%) by echocardiography	30 ± 6.9
	30 (25–35.8)
ACEF score*	2 ± 0.8
	2 (1.4–2.7)
Angina	11 (34 %)
NYHA functional class	
1	0
2	23 (72 %)
3	9 (28 %)
Atrial fibrillation/Atrial flutter	1 (3 %)
Previous MI	18 (56 %)
Previous STEMI	14 (44 %)
Q waves	19 (59 %)
Previous PCI	11 (34 %)
Previous CABG	4 (13 %)
ACEi/ARB	26 (81 %)
Beta blockers	29 (91 %)
Aldosterone receptor antagonists	14 (44 %)
Diuretics	16 (50 %)
Digoxin	1 (3 %)
Statin	30 (94 %)

Values are given as mean ± standard deviation, median (interquartile range) and *n* (%). ACEi indicates angiotensin-converting enzyme inhibitor; *ARB* angiotensin receptor blocker, *CABG* coronary artery bypass graft, *GFR* glomerular filtration rate, *HbA1C* glycosylated hemoglobin, *LVEF* left ventricular ejection fraction, *MI* myocardial infarction, *NYHA* New York Heart Association functional class, *PCI* percutaneous coronary intervention, *STEMI* ST-elevation myocardial infarction

*ACEF score: age (y)/ejection fraction (%) +1 (if serum creatinine >2 mg/dL)

### PCI data

Baseline angiographic characteristics of the 32 patients with successful CTO-PCI are shown in Table 2. Most of the patients had multi-vessel disease with a single CTO. The most common location of occlusions was left anterior descending artery (LAD), followed by right coronary artery (RCA) and left circumflex artery (LCX). A total of 34 CTOs were recanalized, 16 in LAD, 10 in RCA, and 8 in LCX. Within the group of patients with more than one CTO, 7 of these CTO were not recanalized because of a lack of ischemia and myocardial viability in the territory subtended by the occluded vessel, according to study criteria. Two CTO-PCI procedures were required in 4 patients, two of them with retrograde approach. Drug-eluting stents were implanted in 94 % of successful CTO-PCIs, with a mean of 2 ± 1.1 stents/lesion (range 0–5) and a stent length of 47 mm ± 27 (range 0–116). Eleven patients (34 %) underwent non-CTO PCI. Complete anatomical revascularization rate was 91 %. No procedural complications (coronary perforation, cardiac tamponade or emergent cardiac surgery) were observed in any patient undergoing CTO-PCI attempt. No patient died, and none had Q wave myocardial infarction or stroke during the hospital phase. Patients were prescribed aspirin indefinitely and clopidogrel 75 mg daily for at least 12 months after successful CTO-PCI.

**Table 2:** Baseline angiographic characteristics of the study group

	Successful CTO-PCI *n* = 32
Vessels with CTO	41
1	24 (75 %)
2	7 (22 %)
3	1 (3 %)
CTO distribution	41
LAD	17 (41 %)
RCA	15 (37 %)
LCX	9 (22 %)
Coronary arteries with significant stenosis	
1	5 (16 %)
2	19 (59 %)
3	8 (25 %)
Rentop score	1.7 ± 1.3
	2 (0–3)
Syntax score	22.7 ± 10.2
	21.5 (15.5–29.5)

Values are given as mean ± standard deviation, median (interquartile range) and *n* (%). CTO indicates chronic total occlusion; *LAD* left anterior descending artery, *LCX* left circumflex artery, *RCA* right coronary artery

### CMR findings

Myocardial viability in at least two contiguous CTO dependent myocardial segments was present in all the included patients. Only five patients (16 %) had ischemia in two or more myocardial segments subtended by a CTO (mean number of ischemic segments subtended by a CTO in the study population was 0.6 ± 1.4 per patient [range 0–6]).

At 6-month follow-up, CMR studies were performed in 29 patients (two patients refused the repeat CMR and 1 patient suffered sudden cardiac death before follow-up CMR). A significant decrease in LVESV was found (160 ± 54 ml vs. 143 ± 58 ml; *p* = 0.029), along with a 6.4-point improvement in LVEF (31.3 ± 7.4 % vs. 37.7 ± 8 %; *p* < 0.001) (Table 3). No significant changes in LVEDV (230 ± 64 ml vs. 221 ± 58 ml; *p* = 0.25) and necrotic mass (22 ± 12 g vs. 20.4 ± 10.6 g; *p* = 0.13) were seen after successful CTO-PCI. There was no difference in LVEF improvement between the 11 patients (38 %) who underwent associated non-CTO PCI and the 18 patients (62 %) without this intervention (5.9 ± 6.7 [IQR 0–11] vs. 6.9 ± 8.2 [IQR 2–11] points; *p* = 0.73). The improvement in LVEF after successful CTO-PCI persisted in a mixed-effects multivariate model adjusted for baseline ACEF score and non-CTO PCI (*p* < 0.001). The degree of LVEF improvement was not significantly modified by baseline parameters such as LVEF (8.5 ± 8.9 [IQR 2–13.6] vs. 4.4 ± 5 [IQR 0–8.2] points; *p* = 0.14), history of hypertension (4.8 ± 6.4 [IQR 0–11] vs. 9.3 ± 8.3 [IQR 5–13.6] points; *p* = 0.12), hypercholesterolemia (8.3 ± 8.3 [IQR 3–11] vs. 4.8 ± 6.3 [IQR 0.5–8.5] points; *p* = 0.21) or diabetes (5.3 ± 6.7 [IQR 1–11] vs. 8.8 ± 8.5 [IQR 6–9] points; *p* = 0.25), or treatment with angiotensin-converting enzyme inhibitors/angiotensin receptor blockers (7.9 ± 7 [IQR 3–13.6] vs. 6 ± 7.5 [IQR 1–11] points; *p* = 0.57), beta blockers (2.7 ± 6 [IQR 3–9] vs. 6.8 ± 7.4 [IQR 2–11] points; *p* = 0.36) or aldosterone receptor antagonists (5.2 ± 6.2 [IQR 1–9] vs. 8.1 ± 8.7 [IQR 3.5–12] points; *p* = 0.31).

**Table 3:** Comparison of LVEF, LV volumes, LV mass and LV necrotic mass by CMR before and after successful CTO recanalization

	Successful CTO-PCI *n* = 29			
	Pre-PCI	Post-PCI	Difference	*p*
LVEF (%)	Mean 31.3 ± 7.4	Mean 37.7 ± 8	6.4	<0.001
	Median (IQR) 32 (26–37)	Median (IQR) 39 (35–43)		
LVEDV (ml)	Mean 230 ± 64	Mean 221 ± 58	−9.1	0.25
	Median (IQR) 216 (182–257)	Median (IQR) 205 (185–262)		
LVESV (ml)	Mean 160 ± 54	Mean 143 ± 58	−17	0.03
	Median (IQR) 144 (127–194)	Median (IQR) 130 (106–166)		
LV mass (g)	Mean 142 ± 43	Mean 139 ± 47	3.5	0.63
	Median (IQR) 132 (107–164)	Median (IQR) 128 (108–157)		
LV necrotic mass (g)	Mean 22 ± 12	Mean 20.4 ± 10.6	−1.6	0.13
	Median (IQR) 19.2 (13–30.4)	Median (IQR) 18 (12.3–28.7)		

CTO indicates chronic total occlusion; *IQR* interquartile range, *LV* left ventricle, *LVEF* left ventricular ejection fraction, *LVEDV* left ventricular end-diastolic volume, *LVESV* left ventricular end-systolic volume, *PCI* percutaneous coronary intervention

A total of 464 segments were available for perfusion analysis and 493 for regional contractility. The number of segments with normal wall motion or mild/moderate hypokinesia improved after successful CTO-PCI (8.5 ± 4.5 vs. 11.2 ± 3.5; *p* = 0.001) and the number of segments with severe hypokinesia, akinesia, or dyskinesia was significantly reduced (8.3 ± 4.6 vs. 5.7 ± 3.5; *p* = 0.002). The same changes were seen when the analysis was restricted to recanalized CTO-dependent myocardial segments (Table 4). After successful CTO-PCI, there was also a significant reduction in the number of segments showing an inducible perfusion defect in territories subtended by CTOs (0.5 ± 1 vs. 0.2 ± 0.5; *p* = 0.043). In the global analysis including 16 segments per patient, a trend toward a reduction in the number of ischemic segments was maintained (1 ± 1.8 vs. 0.3 ± 0.8; *p* = 0.055).

**Table 4:** Comparison of regional contractility by CMR before and after successful CTO recanalization

		Successful CTO-PCI *n* = 29			
Regional contractility		Pre-PCI	Post-PCI	Difference	*p*
Global	Normal or mild-moderate hypokinesia	8.5 ± 4.5	11.2 ± 3.5	2.7	0.001
	Severe hypokinesia/akinesia/dyskinesia	8.3 ± 4.6	5.7 ± 3.5	−2.6	0.002
CTO- dependent segments	Normal or mild-moderate hypokinesia	2.9 ± 2.2	3.6 ± 2	0.7	0.011
	Severe hypokinesia/akinesia/dyskinesia	3.9 ± 1.8	3.3 ± 1.3	−0.62	0.029

Values are given as mean ± standard deviation. CTO indicates chronic total occlusion; *PCI* percutaneous coronary intervention

### Clinical follow-up

**Fig. 3:**
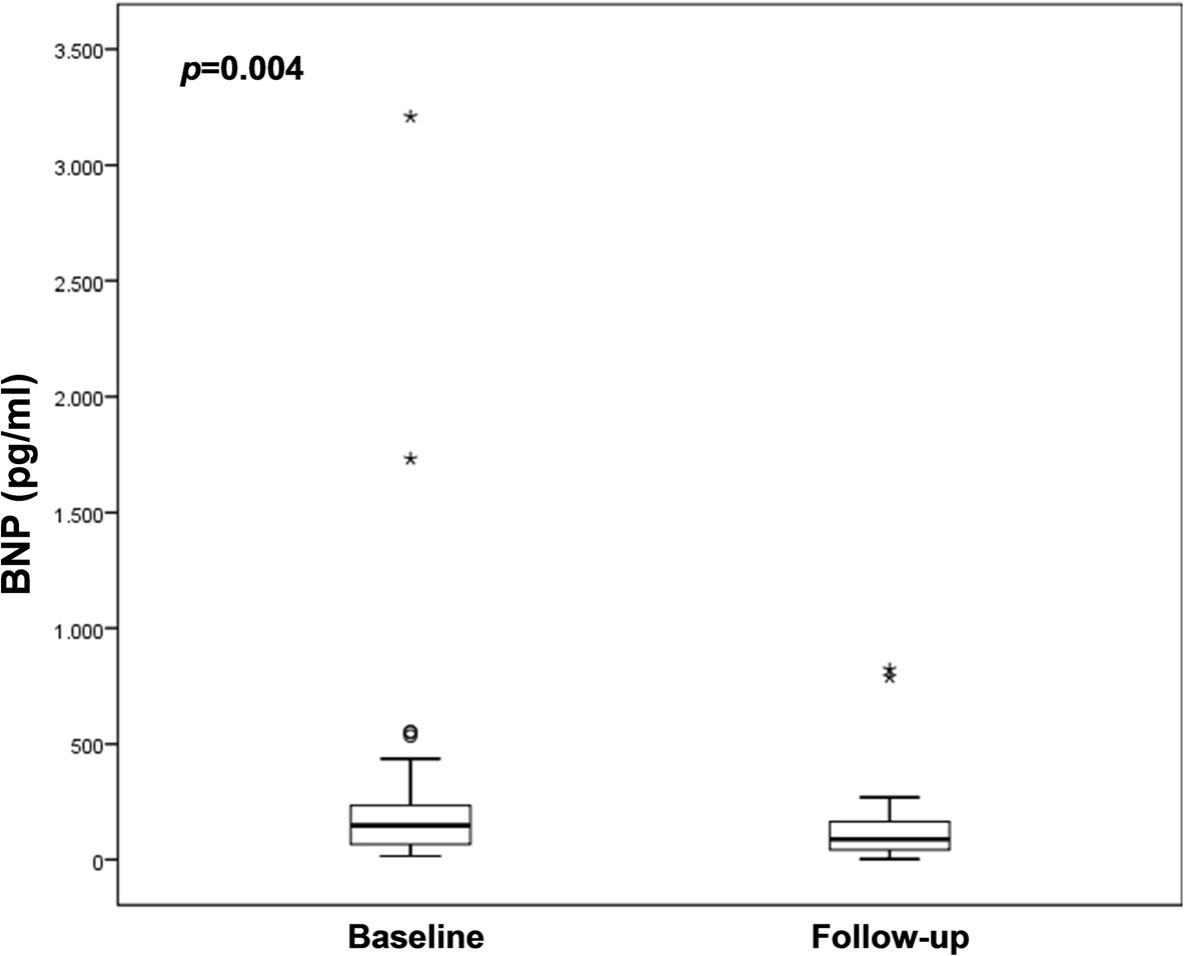
Graph showing a significant reduction in brain natriuretic peptide (BNP) levels after successful CTO-PCI (*n* = 31)

**Fig. 4:**
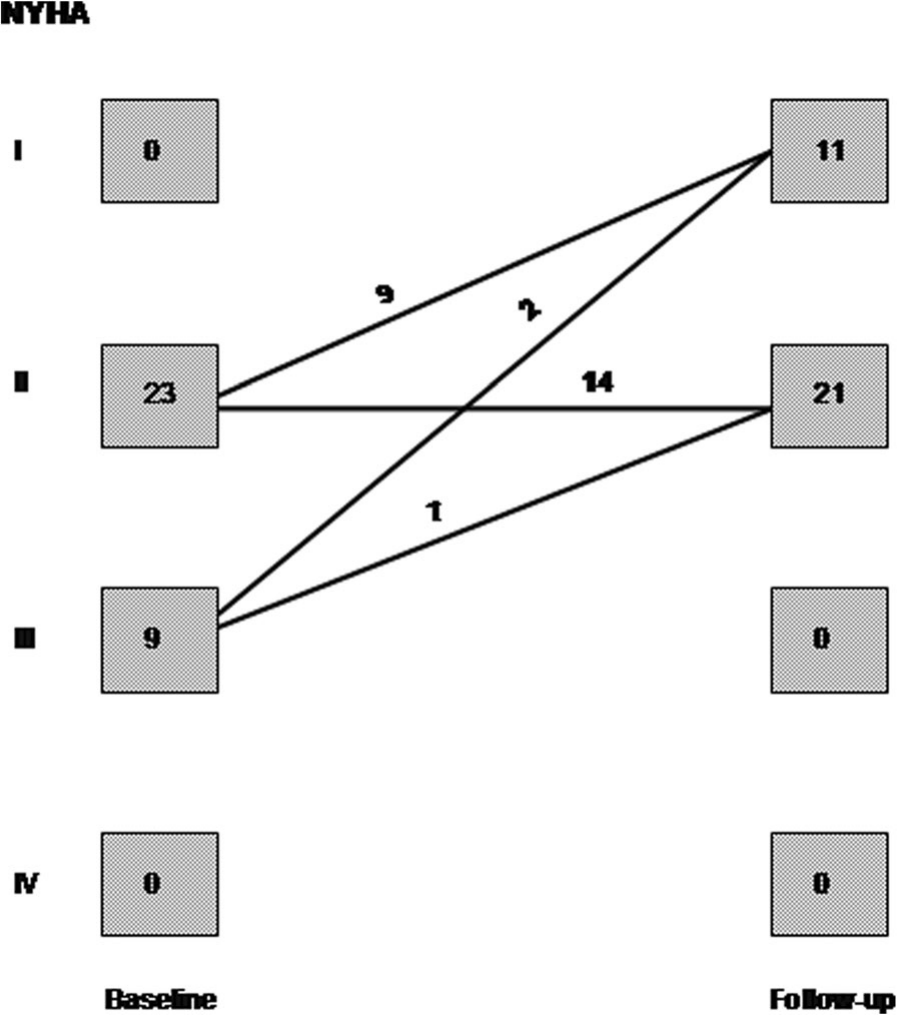
Graph showing changes in New York Heart Association (NYHA) functional class for dyspnea after successful CTO-PCI (*n* = 32)

At 6 months after successful CTO-PCI a significant reduction, compared to baseline, was observed in the proportion of patients with angina (34.4 % vs. 3.1 %; *p* = 0.002) and in BNP levels (323 ± 657 pg/ml [IQR 60.4–238.2] vs. 123 ± 151 pg/ml [IQR 40.6–154.5]; *p* = 0.004) (Fig. 3). Follow-up BNP data were not available in one patient who died 6 months after the procedure and before blood test collection. NYHA functional class for dyspnea improved significantly, with a higher proportion of patients in NYHA I and II at follow-up (72 % vs. 100 %; *p* = 0.004) (Fig. 4).

## Discussion

In this study we show that in a small group of patients with CTO and HFrEF, selected for the presence of viability and/or ischemia in myocardial segments subtended by the occluded vessel by means of CMR study, a significant improvement in LVESV, regional contractility, LVEF and myocardial ischemia was observed after successful CTO-PCI.

From a clinical point of view, an improvement in angina and NYHA functional class, along with a decrease in BNP levels was seen after CTO recanalization.

To our knowledge, this is the first study to date evaluating the benefits of CTO-PCI in patients with HFrEF. The proportion of patients with history of previous myocardial infarction and the high prevalence of classic cardiovascular risk factors in this cohort are consistent with previous published data [22, 23]. Improvement in angina status after CTO-PCI has also been shown in previous studies in patients with preserved LVEF [7, 8, 12, 16, 18].

CMR is a powerful tool over other modalities to assess viability in patients with reduced LVEF being considered for CTO-PCI and to determine improvement in LVEF after successful CTO-PCI in this population. The CMR findings in our study partially correlate with those reported by Baks et al. and Kirschbaum et al. [9, 10] Both of these previous studies, with sample sizes similar to our cohort, showed a reduction in LVESV and LVEDV with no significant improvement in LVEF after successful CTO-PCI. It should be noted, however, that inclusion criteria in both studies differed from our cohort: the viability of CTO-dependent myocardial segments assessed by CMR was not a criterion, and all patients had preserved LVEF. All cases in our cohort had LVEF ≤40 % and showed evidence of viability by CMR in myocardial segments subtended by a CTO. In 17 patients with well-preserved LVEF, Cheng et al. [11] also reported improvement in wall thickening of myocardial segments subtended by a successfully recanalized CTO and no changes in LVEF and LV volumes by CMR. All three mentioned studies [9–11] showed that the improvement in regional wall thickening after a successful CTO-PCI was indirectly related to the transmurality of LGE in a myocardial segment. More recently and in consonance with our findings, Bucciarelli-Ducci et al. [12] reported a significant improvement in LVESV and LVEF after successful CTO-PCI in a group of 32 patients with preserved LVEF showing CMR evidence of myocardial viability and ischemia. Other than older studies assessing LVEF with contrast ventriculography [13, 14], Valenti et al. published the only large study to date showing improved LVEF after successful CTO recanalization [15]. In their study, 290 patients with viable myocardium in territories supplied by CTO and most of them with preserved LVEF were evaluated by Doppler echocardiography before and after the PCI procedure.

One third of our patients with successful CTO-PCI also underwent PCI of non-occlusive coronary stenosis. No differences in the degree of LVEF improvement were seen when this group was compared with patients who did not undergo non-CTO PCI, suggesting that changes in LV function parameters after successful CTO-PCI were derived from CTO recanalization. Baseline patient characteristics such as LVEF, targeted therapies for HFrEF and the presence of cardiovascular risk factors did not have a significant impact on the degree of LVEF improvement after CTO-PCI.

Improvement in myocardial ischemia assessed by CMR adenosine stress perfusion imaging after successful CTO-PCI has been previously demonstrated in small series. Pujadas et al. [7] showed a reduction in the number of ischemic segments in 33 patients who underwent successful PCI of a single CTO. Similarly, Bucciarelli-Ducci et al. [12] described a complete or almost complete resolution of perfusion defect after CTO-PCI along with an increased myocardial perfusion reserve in the CTO territory. It is worth mentioning that in both studies LVEF was preserved in most of the patients before the PCI procedure and mean necrotic mass was much lower than in the present study (6 and 11 g vs. 22 g).

Interestingly, changes in CMR parameters after successful CTO-PCI were associated with an improvement in NYHA functional class for dyspnea in our study, along with a significant reduction in BNP levels. These clinical outcomes had not been previously evaluated in the CTO-PCI field. Along with LVEF and NYHA functional class, BNP levels are a prognostic marker in the population with HFrEF and they can be a useful clinical tool to stratify the risk of adverse events in this population [24, 25]. Improvement in ischemia burden, LVEF, NYHA functional class and BNP levels after CTO-PCI in patients with HFrEF could lead to improved prognosis in this population and provide a rationale for attempting CTO recanalization after viability and/or ischemia confirmation in the territory subtended by the occluded vessel.

Finally, the absence of procedural complications or any increase in the amount of myocardial necrosis in the present study may suggest that the CTO-PCI procedure is safe in selected patients with HFrEF.

This study has several limitations worthy of mention. The first and main limitation is that this was a non-randomized study without a predefined control group; therefore, changes in LV function and volumes or in clinical parameters cannot directly be attributed to the CTO-PCI procedure, but may be related to conservative therapy. The second major limitation is the small sample size. Third, given the strict patient selection, the study may not be representative of the whole population with CTO and HFrEF. Finally, we did not perform a second angiography at 6 months after PCI in any of the patients. The major strength of this study is that it is the first report of CMR assessment of LV function after CTO-PCI to focus specifically on the important population of patients with HFrEF. The results derived from this study should be interpreted cautiously and prospective randomized controlled trials are warranted to validate these findings with more reliable clinical evidence.

## Conclusions

In this group of patients with HFrEF showing CMR evidence of viability and/or ischemia within the territory supplied by the CTO, an improvement in ejection fraction, left ventricular end-systolic volume and ischemia burden was observed after CTO-PCI. Clinical and laboratory parameters also improved.

## Abbreviations

BNP: Brain natriuretic peptide
CMR: Cardiovascular magnetic resonance
CTO: Chronic total occlusion
HFrEF: Heart failure with reduced ejection fraction
LAD: Left anterior descending artery
LCX: Left circumflex artery
LGE: Late gadolinium-enhancement
LV: Left ventricle
LVEDV: Left ventricular end-diastolic volume
LVEF: Left ventricular ejection fraction
LVESV: Left ventricular end-systolic volume
NYHA: New York Heart Association
PCI: Percutaneous coronary intervention
RCA: Right coronary artery
